# The prevalence of intimate partner violence among couples signing up for universally offered parent preparation

**DOI:** 10.1371/journal.pone.0223824

**Published:** 2019-10-15

**Authors:** Tea L. Trillingsgaard, Hanne N. Fentz, Marianne Simonsen, Richard E. Heyman

**Affiliations:** 1 Department of Psychology and Behavioural Sciences, Aarhus University, Aarhus, Denmark; 2 Trygfonden’s Center for Child Research, Aarhus, Denmark; 3 Department of Economics and Business Economics, Aarhus University, Aarhus, Denmark; 4 Family Translational Research Group, New York University, New York, United States of America; Arizona State University, UNITED STATES

## Abstract

**Background:**

Intimate partner violence (IPV) during pregnancy can have serious health consequences for mothers and the unborn child. Nevertheless, IPV is seldom addressed in the context of parent preparation.

**Aim:**

This study aimed to map the prevalence, direction, and severity of IPV in a sample of expectant couples signing up for universally-offered parent preparation.

**Method:**

A total of 1726 Danish couples expecting their first child provided data on physical and psychological IPV by completing the Family Maltreatment measure during the second trimester of pregnancy.

**Results:**

In 18.5% of the couples, at least one partner reported psychological or physical IPV acts during the past year. In more than 8% of couples, one or both partners reported acts and impacts above the ICD-11 threshold for clinically-significant IPV (CS-IPV) during the past year (3.6% physical CS-IPV, 5.3% psychological CS-IPV, and 0.8% both physical and psychological CS-IPV). Among couples with physical IPV below the clinical threshold, pregnant-woman-to-partner (50%) and bidirectional (38.2%) IPV were more common than partner-to-pregnant-woman IPV (11.8%). Among couples with physical CS-IPV, pregnant-woman-to-partner (36.1%), partner-to-pregnant-women (29.1%) and bidirectional (34.4%) forms were equally common. Among couples with psychological IPV, pregnant-woman-to-partner (54.9%) and partner-to-pregnant-woman (39.6%) IPV were more common than bidirectional IPV (5.5%).

**Discussion:**

The prevalence of violence was markedly higher in this study compared with previous reports from the Nordic region and highlights a previous oversight of a substantial and clinically significant level of pregnant-woman-to-partner IPV—as well as the reverse. Data from this study call for IPV to be addressed in universally offered parent preparation programs.

## Introduction

Prevention of intimate partner violence (IPV) is an important public health goal across the globe [1]. For both women and men, IPV victimization increases risk of physical injury, of poor health, depressive symptoms, substance abuse, developing a chronic disease, mental illness, or injury [2]. The detection of IPV during pregnancy is of special concern because adverse effects of IPV include preterm delivery, low infant birth weight, postnatal maternal depression, and maternal suicidal thoughts and attempts, for reviews see [3–5].

During the transition to parenthood, new responsibilities and care-taking demands emerge, accompanied by both joy and parental fatigue [6]. Time for leisure and couple intimacy is reduced [7] and more rapid declines in relationship functioning tend to occur among new parents [8]. Studies on IPV toward women have found both increases and decreases of IPV during pregnancy with some indication that pregnancy may be a protective factor for physical IPV but a risk factor for psychological IPV [7]. The combination of increased psychosocial demand and parent motivation to protect and build a healthy family highlights this time period as particularly meaningful for prevention.

Promising outcome data on approaches to manage severe IPV during pregnancy do exist, including data on emergency response systems (such as trauma center housing, advocacy, and safety planning) as well as longer-term treatments; for reviews see [9–11]. Although these approaches are clearly important, a comprehensive public health approach to IPV also necessitates the development of effective prevention programs that reach couples before more severe or harmful IPV occurs [12].

Primary IPV prevention during the neonatal period has been identified as particularly favorable [12], given couples’ increased openness to learning/improving relationship and parenting skills [13], high-risk couples’ greater likelihood in participating in neonatal, versus premarital, prevention [14] and the increase in IPV risk associated with the almost inevitable decline in relationship satisfaction following the birth of a child [15, 16].

Programming for primary IPV prevention during this period is not well developed. Feinberg and colleagues have found reductions in psychological [17] and physical [18] IPV perpetration from the Family Foundations program for expectant first-time parents, comprising five 3-hour prenatal group sessions and four 2-hour postnatal group sessions. The Family Foundations program targets factors associated with IPV (e.g., communication), but does not target IPV directly. Bair-Merrit and colleagues [19] found a decrease in frequency of physical IPV victimization in mothers who received a three-year home visitation program; however, occurrence (yes/no) of physical or psychological IPV did not differ between program mothers and controls. Alternatively, Heyman and colleagues [12] did not find impacts on physical or psychological IPV using the Couple CARE for Parents program (participants did not have to be first-time parents) comprising eight 1-hour postnatal sessions with individual couples; further, this study reported indications that physical IPV at post-treatment was less likely for those with planned pregnancies but higher for those with unplanned pregnancies. Similarly, one of the largest, best-powered studies in this area—the evaluation of the U.S. Building Strong Families program with low-income, unmarried, predominantly racial/ethnic minority parents of newborns—pooled data across eight sites and found no differences between couples who received skills-based prevention and no-intervention control couples on relationship outcomes (e.g., satisfaction) or on IPV at the post-program assessment [20].

Also, in the Nordic countries, many resources are invested in prevention programs targeting new or expectant parents and universal approaches are increasingly common. As an example, 63% of Danish municipalities currently offer parenting programs that are routinely offered to all first-time parents on topics such as delivery, finances, feeding the child, roles and family dynamics, and sleeping patterns [21]. A review of the teacher manuals from the three most widespread programs [22–24] revealed that IPV is not directly addressed in any of them. This may have several explanations. One is that planners of universal programs may consider IPV to be an issue belonging to specialized treatment, not broad-based prevention. Another is the (mis)conception, even among professionals, that acts of IPV are rare in couples participating in prevention programs. Yet another is that couple conflicts escalating to IPV may be perceived by developers as difficult to bring up in a group setting, given the sensitive and stigmatized nature of IPV. Finally, IPV may be thought of as an aversive or even dangerous topic to address directly. This position has been taken by some scholars and jurisdictions who prohibit treating IPV in the presence of male partners [25, 26]. Guidelines for addressing IPV in the context of universal parenting program are missing from the literature. As we will review below, this may perhaps be explained by the scarcity of reliable data on the occurrence of IPV in these samples.

### The prevalence of IPV in Nordic countries

According to a 2014 survey conducted across the 28 European Union (EU) member states by the EU Agency for Fundamental rights [27], the mean lifetime prevalence of physical and/or sexual IPV victimization of women in Europe was 22% but with comparably higher rates among Nordic countries, including Denmark (32%), Finland (30%), and Sweden (28%). For psychological IPV (by current or previous partner), Nordic countries ranked similarly high, with Denmark (60%), Finland (53%), and Sweden (51%) ranking substantially higher than the EU average (43%).

Nordic data on violence against pregnant women provides useful context. A large cohort study among pregnant women in Norway found that 5% reported some type of violence during the past year from any type of perpetrator (including that from strangers; [28]). In line with this result, a study of pregnant women in Sweden found a past-year prevalence of 4.3% for any type of violence, with 3.1% for emotional, 1.9% for physical, and 0.1% for sexual violence across any type of perpetrator [29]. A survey across nine obstetric departments in Denmark collected data on physical and sexual violence against pregnant women from any perpetrator, yet only lifetime prevalence was obtained: 16.1% for moderate physical violence, 9.9% for severe physical violence, and 9.2% for sexual violence [30]. In one additional study including data from Denmark, a prevalence rate of 1.8% for physical violence from an intimate partner during pregnancy was found [31]. This rate was based on a secondary analysis of data from the International Violence Against Women Survey, in which women from 19 different countries and at different life stages were interviewed via telephone and asked to recall whether IPV had occurred during previous pregnancies.

However, methodological issues limit the conclusions to be drawn about IPV in these Nordic pregnant samples. The Norwegian study asked an exceedingly broad question, which, instead of inquiring about specific behaviors, required women to make inferences (e.g., “have you been subjected to physical abuse”). The Danish study asked only about lifetime occurrence (e.g., “have you ever been pushed, shoved or slapped?”). With the exception of one [31], no studies differentiated IPV from violence by other perpetrators (strangers, other family members). Furthermore, existing Nordic studies used operationalizations of severity similar to those of the Conflict Tactics Scales [32], that is, severity as delineated by a priori notions of seriousness of acts rather than by impacts suffered. For example, the NorVold Abuse Questionnaire [33] defines hitting as mild, pushing as moderate, and showing a weapon as severe violence. This is problematic because the same type of act can vary in impact severity (e.g., consequences of pushing someone away versus pushing someone down the stairs). Finally, none of the existing Nordic studies examine IPV perpetration by pregnant women toward partners, thus focusing exclusively on the pregnant mother as a victim. This is common in IPV prevalence studies with pregnant and other samples. However, results from a home-visiting program in the Netherlands targeting mothers at high risk of IPV victimization found that this group of women were often both victims and perpetrators of IPV and that their perpetration also led to clinically significant harm to their partners [34].

In sum, the latest European survey indicates that Nordic countries, compared with other European countries, have higher lifetime rates of IPV toward women; this deviates from the relatively low rates of violence found in previous Nordic studies with pregnant samples. Because of differences in assessment (e.g., questions, time span) and participants (all women versus pregnant women), it is difficult to make firm conclusions. Thus, to guide prevention, further research is clearly needed on the prevalence, type, direction, and severity of IPV in couples expecting a child.

### Study aims

To address the above shortcomings, we used anonymous, detailed reports of IPV from both partners and a validated measure operationalizing DSM-5 and ICD-11 criteria for clinically significant (CS) physical and psychological IPV. This study investigated the one-year prevalence, type (psychological and/or physical), severity (non-impactful acts versus those meeting ICD-11 clinically significant guidelines [35]), and direction of IPV (partner→pregnant woman, pregnant woman→partner, or bidirectional) in a sample of pregnant women and their partners who signed up for a universally offered parent preparation program.

## Materials and methods

The research described has been approved by the Regional Ethical Committee (Central Denmark Region); registration number ESDH 1-10-72-109-14, approved by the Danish data protection agency; registration number 2014-41-3016. Written and oral consent was obtained from all participating subjects.

### Participants

This study included 1726 couples in which at least one partner completed an anonymous questionnaire on IPV (1616 couples with both partners completing, 85 couples with only the pregnant woman completing, and 25 couples with only the partner completing). All study participants were enrolled in a randomized controlled trial [36] of the Family Startup Program, a universally offered pre- and postnatal parent preparation intervention [22]. The overall aim of the program is to enhance parental sense of confidence and lower parental stress by increasing father involvement and providing better access to sources of professional and informal network support. The Family Startup Program includes 12 group meetings with 6–9 families in each group. Physical or psychological IPV is not addressed directly in the curriculum.

Pregnant women were eligible if (a) living in Aarhus Municipality, Denmark, (b) being in a romantic relationship, and (c) expecting their first child. Romantic partners to pregnant women were eligible regardless of their biological or legislative relationship to the child. Both partners were excluded if (d) either partner was under the age of 18 years, (e) incapable of managing his/her own legal affairs, or (f) did not have sufficient Danish skills to understand information about the project or to take part in the Family Startup Program. Further, women identified as needing specialized family, drug, or alcohol abuse treatment by their general practitioners or the prenatal unit (g) were referred to other services prior to our recruitment. Standard procedures for identifying vulnerability in the family did not include screeners for physical or psychological IPV.

Pregnant participants were on average 29.4 years old (SD = 3.5) and partners 31.2 years old (SD = 4.75) at the time of delivery. Among partners, 1617 cases were biological fathers and 24 were same sex partners. Due to the recruitment during prenatal visits, all same sex couples in the study were female. To distinguish members of the couple, we consistently use the terms *pregnant woman* and *partner* even though their reports on past-year IPV include both approximentally four months of pregnancy and approximentally eight months before. These terms do not imply that the reported IPV only or necessarily occurred during pregnancy. Average relationship length was 4.5 years (*SD* = 3.2), 26% were married, and 94% cohabitating. Data on age and civil status were obtained from registers on families of children born after January 1, 2017. The municipality of Aarhus—the second largest city in Denmark with approximately 275,000 inhabitants—covers a metropolitan area, a number of smaller suburban towns, as well as rural areas. In 3% of the families, Danish was not the preferred language at home; the most common other languages were English, German and Spanish. Most participants were employed (*n* = 2430, 72.7%); the largest subgroup without employment were students (*n* = 728, 21.8%). Financial strain (e.g., not being able to pay the bills) was a perceived issue reported by at least one partner in 270 couples (15.6%).

### Procedures

#### Recruitment

Couples were recruited at the prenatal unit of the Department of Obstetrics and Gynaecology, Aarhus University Hospital, from November 2013–December 2016. In Aarhus municipality, general practitioners refer all pregnant women for ultrasound imaging at this unit. All referrals were screened for study eligibility, and mothers who fulfilled the criteria were informed about the study through an e-mail from the hospital with links to more information. Both partners were informed and recruited for the study when at the ultrasound appointment during week 12 or 19 of the pregnancy. If couples needed more time or information, they received a telephone call from the recruitment team one of the following days. According to hospital statistics, more than 99 percent of all pregnant mothers in the municipality attend the ultrasound appointment. A total of 3615 mothers and their partners were screened for eligibility; 163 did not meet eligibility criteria, 1494 actively declined the invitation to participate, and 177 did not respond to recruitment calls. A total of 1781 mothers (with or without partners) signed up for the Family Startup project and were randomized. Among these, we received a questionnaire response on the Family Maltreatment measure from at least one partner in 1726 couples, constituting a response rate of 97%. The use of register-based data allowed us to compare participants in the study with the population of first-time parents in Aarhus Municipality in 2014, and this comparison is presented in the supporting information file (S1 Table). Socioeconomically, the study sample was comparable to the population overall, with study participants (on average) being one year younger and earning somewhat lower work salary. Compared with the population, the study sample included fewer immigrants (likely due to exclusion criteria f) and fewer mothers and fathers with previous psychiatric contacts (due to exclusion criteria g).

#### Data collection

After signing up, but prior to randomization, an e-mail was sent to each partner with a personal login to the web-based survey. Partners were instructed to fill in the questionnaires separately. Two reminders were sent by e-mail. Anonymity was ensured so that neither researchers nor program leaders were able to link participant identity and questionnaire data. No monetary incentives were used to motivate participation. A lottery prize with a value of maximum DKK 3,000 (approximately $470) were drawn from the pool of those who filled in the questionnaire.

#### Measures

Background information about gender, relationship length, employment status, smoking, language preference in the home, place of birth, family of origin stability, and financial strain was collected through single items in the online questionnaire. Information on parental age, marital status, and educational background was drawn from registers.

Intimate Partner Violence was measured with the Family Maltreatment measure [37], which comprises questions tailored to the DSM-5 and ICD-11 criteria for clinically significant physical and psychological IPV (CS-IPV). Physical CS-IPV refers to any nonaccidental act of physical force that (a) results in, or has more than reasonable potential to result in, physical harm to an intimate partner, or (b) evokes significant fear in the partner. Psychological CS-IPV refers to any nonaccidental verbal or symbolic act that results in significant psychological harm to an intimate partner. Psychological harm includes significant fear, significant psychological distress, somatic symptoms that interfere with normal functioning, and fear of IPV reoccurrence that causes the victim to significantly limit activities in five major life areas (work, education, religion, medical or mental health contacts, contact with friends/family). For this study, we used the term low impact (LI) physical IPV to refer to any nonaccidental act of physical force that does not meet the CS-IPV threshold. The translation of items into Danish was conducted by the first and the second author independently; disagreements were first discussed internally then with one of the developers of the measure (Dr. Heyman). All Danish items were then reviewed by a Danish expert within IPV research, Dr. Bramsen, to reach the final version.

Physical LI-IPV and CS-IPV were measured as follows: Respondents completed 30 items arranged in 15-item pairs assessing the frequency of IPV acts perpetrated by themselves and their partners in the previous year [37]. Acts were generally similar to those in the Physical Assault Subscale of the Revised Conflict Tactics Scales [32]. One item allowed respondents to indicate “other” and write in a particular act not listed. These acts were coded without disagreement by the first author and an assisting coder as IPV or not IPV. The majority of write-in responses (52%) did not qualify as IPV and were easily differentiated (e.g., “slammed a door” versus “slammed a door against my partner”). If an act was reported, fifteen follow-up questions asked about injuries resulting from each act. As noted above, physical CS-IPV was operationalized as reporting physically aggressive acts of IPV with an impact (e.g., reporting physical injury, endorsing an act of high inherent dangerousness, or victim reporting fear). Data on the frequency of IPV were obtained (e.g., the following item “during the past 12 months, how many times did your partner push or shove you,” had response categories of “never”,” once”, “twice”, “3–5 times”, “6 times or more”). We calculated the between-partners consistency in reports of any occurrence of partner-to-pregnant-woman and pregnant-woman-to-partner physical LI-IPV and CS-IPV. Because past research indicates that, in community samples, both men and women under-report IPV versus couple-level reports (e.g. [38]), we used any self- or partner-reported IPV regardless of intradyadic consistency to determine the occurrence of physical LI-IPV and CS-IPV in a couple.

Psychological CS-IPV was measured as follows: All participants filled in screener items asking for experiences of significant depression, stress, and/or fear caused by their partners’ behavior. If this was endorsed, respondents were presented with two lists of potential other psychological abuse impacts (a) fear of harm to self or close others and (b) fear that significantly interfered with the victim’s ability to carry out any of five major life activities (e.g., “contact my family or friends”, “get a job or pursue a career”). Then, 10 specific acts were presented and respondents were asked how often (in the past year) their partners had committed them (e.g., “put me down or humiliated me,” “stalked me,” “grilled or interrogated me about where I had been, what I had done, etc.”). If at least one act was reported in the past year, participants were presented with a list of all endorsed acts and were asked whether the acts had caused or contributed to the interfering depression, stress, and/or fear that they had reported earlier. Data on frequency were obtained (e.g., the following item “during the past 12 months, how many times did your partner push or shove you,” had response categories of “never”,” once”, “twice”, “3–5 times”, “6 times or more”). Psychological CS-IPV victimization was operationalized as at least one reported act of psychological IPV that caused either (a) significant fear, stress, depression, (b) fear of own or close others’ safety or (c) fear that interfered with the pursuit of major life activities.

#### Data analyses

**Fig 1:**
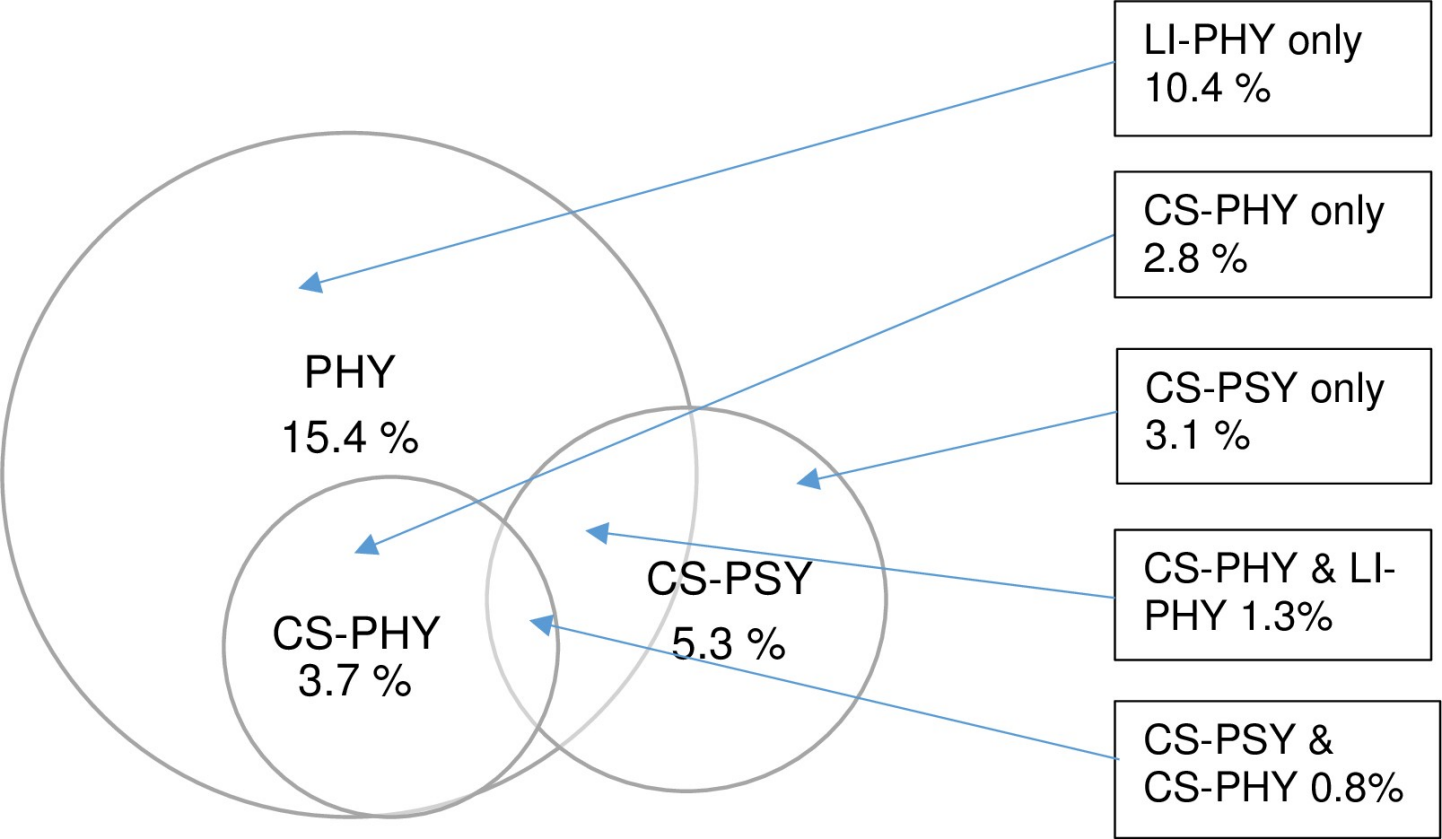
The prevalence of IPV (during the past year) by co-occuring types. PHY = any acts of physical IPV. LI-PHY = physical IPV below the level of clinical significance. CS-PHY = physical IPV above the level of clinical significance. CS-PSY = psychological IPV above the level of clinical significance.

To describe IPV prevalence at the couple level, we classified each couple into the following six mutually exclusive categories: (1) *No IPV*, if both of partners reported no acts of physical IPV and did not meet the criteria for psychological CS-IPV; (2) *Physical LI-IPV-only*, if (a) one or both partners reported any past-year physical IPV acts and (b) neither partner reported impact from physical or psychological IPV; (3) *Physical CS-IPV-only*, if one or both partners reported physical IPV with impact and neither partner reported any psychological CS-IPV; (4) *Psychological CS-IPV-only*, if one or both partners reported both psychological IPV and impact during the past year, and neither partner reported any physical IPV; (5) *Psychological CS-IPV & Physical LI-IPV*, if both physical and psychological IPV were reported by at least one partner but only psychological IPV had impact; and (6) *Psychological CS-IPV & Physical CS-IPV*, if both impactful psychological and impactful physical IPV were reported during the past year from one or both partners. To describe the prevalence of the overall, yet sometimes co-occurring types of IPV, we combined (3) & (6) into physical CS-IPV prevalence, (2), (3), (5), & (6) into physical IPV prevalence, and (4), (5), & (6) into psychological CS-IPV prevalence. Fig 1 provides these prevalence rates and visualizes the overlapping classifications of physical LI-IPV, physical CS-IPV and psychological CS-IPV.

To describe the direction of physical and psychological IPV, we classified couples into three mutually exclusive categories: (1) pregnant-woman-to-partner IPV, (2) partner-to-pregnant-woman IPV, and (3) bidirectional IPV. This was done separately for physical LI-IPV, physical CS-IPV and psychological CS-IPV, and at the couple level. This means that if the same type of physical LI-IPV (i.e., the partner pushed the pregnant woman 3–5 times) was reported by both the victim and the perpetrator, this would only count as one incident at the couple level. Psychological CS-IPV was based solely on victim reports, which means that each type of act could only include one individual response. We also analyzed repeated IPV by classifying couples as (a) those having up to one act per partner and (b) those reporting more than this. We compared the rates of pregnant victimization and partner victimization using chi-squared tests. This was done on the level of single acts and impacts as well as between groups (pregnant woman victimization, partner victimization, and bidirectional).

To investigate the concordance of partners’ report on physical IPV, we counted the number of cases with and without physical IPV, based on pregnant-woman reports and partner reports. Note that two partner reports were available for 1616 couples, while in 110 couples there were only one respondent and thus they were left out of this analysis.

## Results

As shown in Table 1, 18.5% of the sample (*n =* 613 couples) reported some type of IPV. A total of 15.4% reported some type of physical IPV, with 10.4% reporting only physical LI-IPV (i.e., without clinically significant harm). Psychological and physical IPV occurred concurrently some couples and, as shown in Fig 1, physical and psychological CS-IPV had past year prevalences of 3.7% and 5.3%, respectively. Both physical and psychological CS-IPV were reported by 0.8% of couples for the past year.

**Table 1:** Prevalence of past-year IPV by subtype (*N* = 1726 couples).

	Couples (n)	Percent of full sample
No IPV	1407	81.5
Any IPV	319	18.5
**Subtype**		
Low impact physical IPV-only	181	10.4
Clinically significant physical IPV-only	47	2.8
Clinically significant psychological IPV-only	54	3.1
Clinically significant psychological IPV & Low impact physical IPV	23	1.3
Clinically significant psychological IPV & Clinically significant physical IPV	14	.8

Clinical significance is classified according to ICD-11 diagnostic criteria. Each couple was classified in the most severe applicable category only.

Some couples, especially those with IPV, may hesitate to fill in questionnaires about their relationships. In 3% of the sample (*n* = 55 couples), reports were completely missing from both partners and these couples are not included in Table 1. Under the extreme assumption that all these couples experienced some type of IPV, the prevalence of any IPV during the past year would raise to 21%. In 6% of the sample (*n* = 110 couples), the report from one partner was missing. Due to the nature of the measure, a report from one partner includes both the victimization and perpetration of physical IPV and the perpetration of psychological IPV on the part of the other partner. Thus, couple-level data were included in the analyses from couples with one missing report. In this subsample, the prevalence of IPV (any type) based on one report was 24.5% (27 couples out of 110). Although this proportion is not significantly different from the subsample with two reports (18%; 293 couples out of 1616), there is reason to suspect that some IPV is hidden in the missing responses. In the extreme case that all couples with one or two missing reports (110 with one and 55 couples with two) experienced some type of IPV, the prevalence of IPV (any type) would rise to 24.2% percent for the full sample.

The type, direction, and severity of physical IPV are shown in Table 2. For physical LI-IPV, pregnant-woman-to-partner IPV was significantly more prevalent (*n* = 102 couples, 50% of couples reporting it) than bi-directional IPV (78 couples, 38.2%) which was significantly more prevalent than partner-to-pregnant-woman IPV (24 couples, 11.8%). The most common acts of LI-IPV were pushing, shoving, grabbing, and slapping. For physical CS-IPV, pregnant-woman-to-partner (22 couples, 36.1% of couples reporting it), partner-to-pregnant-woman (18 couples, 29.5%), and bidirectional (21 couples, 34.4%) were approximately equal. The most common acts among those reporting CS-IPV were also pushing or shoving, grabbing, and slapping; the most common impacts were bruises or welts, grazes or wounds, and feeling pain at least 4 hours after. Fear for safety was reported by 15 (5.6% of) couples with physical IPV (13 pregnant reports and 4–5 partner reports).

**Table 2:** The type, direction and severity of physical IPV during the past year.

	Low impact physical IPV (n = 204)				Clinically significant physical IPV (n = 61)			
	One way		Bidirectional		One way		Bidirectional	
	Pregnant vic.	Partner vic.	Pregnant vic.	Partner	Pregnant vic.	Partner vic.	Pregnant vic.	Partner vic.
Number of couples (%) Type of Acts	**24 (11.8)**	**102 (50)**	**78 (38.2)**		18 (29.5)	22 (36.1)	21 (34.4)	
Pushed or shoved	**11**	**80**	50	57	13	13	15	19
Grabbed	15	27	48	40	14	9	20	19
Scratched	-	3	-	**7**	**<3**	**12**	**4**	**13**
Slapped	**<3**	**21**	**9**	**23**	5	6	13	15
Thrown hard object	**-**	**8**	4	9	<3	3	6	6
Bitten	-	<3	3	7	<3	5	3	4
Hit or punched	**-**	**14**	**4**	**12**	<3	5	10	12
Slammed against a wall	-	<3	4	<3	<3	<3	7	5
Held down (e.g. twisted arm, hair)	<3	<3	7	3	**9**	**<3**	11	6
Kicked	-	<3	<3	4	<3	<3	4	6
Choked	-	-	-	-	-	-	<3	-
Hit with an object that could hurt	-	-	-	-	-	-	<3	<3
Other	<3	-	-	-	3	<3	<3	<3
Type of Impacts								
Feared for own safety	-	-	-	-	8	<3	5	3
Felt pain at least 4 hours after	-	-	-	-	5	5	9	6
Graze or wound	-	-	-	-	4	10	6	10
Bruise or welt	-	-	-	-	9	14	19	18
Sprain or fracture	-	-	-	-	-	-	-	-
Fainted	-	-	-	-	-	-	-	-

Note. All numbers refer to couples. One count of act or impact can include reports from either one or two individuals within the same couple. **Bolded numbers indicate a significant difference (p > 0.05) between the number of reports on pregnant and partner victimizations.**

The direction and severity of psychological CS-IPV are shown in Table 3. Pregnant-woman-to-partner psychological CS-IPV (50 couples, 54.9% of those reporting it) was significantly more common than partner-to-pregnant-woman psychological CS-IPV (36 couples, 39.6%), which was significantly more common than bidirectional psychological CS-IPV (5 couples, 5.5%). Across these types, the most prevalent acts were being grilled or interrogated, put down or humiliated, insulted, or sworn at. The most commonly consequence of psychological CS-IPV was feeling so sad, down, or depressed that it affected [oneself] almost every day for more than two weeks. Fear for safety was reported by 12 (13.2% of) couples with psychological CS-IPV (4 pregnant reports and 9 partner reports).

**Table 3:** The type, direction, and severity of psychological IPV during the past year.

	One way (n = 86)		Bidirectional (n = 5)	
	Pregnant vic.	Partner vic.	Pregnant vic.	Partner vic.
Acts/Number of couples (%)	**36 (39.6)**	**50 (54.9)**	5 (5.5)	
Not allowed to have ID, driver’s license or passport	-	-	-	<3
Put down or humiliated	21	27	3	3
Kept from seeing service providers	-	-	-	<3
Stalked	-	<3	-	<3
Not allowed to see/talk to a family member or friend	<3	5	<3	<3
Tried to make me think that I was crazy	**10**	**<3**	3	3
Insulted or sworn at	20	28	5	3
Not allowed access to money	<3	<3	-	<3
Grilled or interrogated	**7**	**35**	<3	3
Impacts				
**Fear of safety**				
…for my own safety due to something my partner did or said	<3	5	<3	<3
… that my partner might physically hurt someone I care about	**-**	**3**	<3	<3
**Human rights**–fear that interfered with…				
…working or pursuing work goals	**-**	**8**	-	<3
… going to school or pursuing educational goals	-	<3	-	<3
… practicing religion or spiritual beliefs	-	-	-	-
… getting the necessary medical or mental health service	-	<3	<3	<3
… contacting family or friends	**-**	**7**	<3	<3
**Emotional distress**				
Depression	20	22	4	<3
Stress	16	20	<3	4
Fear	**-**	**4**	-	<3

Note. Numbers refer to couples. Couples were classified based on victim report only. **Bolded** numbers indicate a significant difference (p > 0.05) between the number of reports on pregnant and partner victimization.

The majority (*n* = 185 couples; 69.8%) of couples with physical IPV had repeated IPV (i.e., more than one act per partner in the last year). Only those reporting psychological impacts were asked about behavioral frequency, so the analyses for psychological IPV only focus on CS-IPV. About three-quarters (*n* = 70 couples; 76.9%) of couples reporting psychological CS-IPV had repeated CS-IPV.

**Fig 2:**
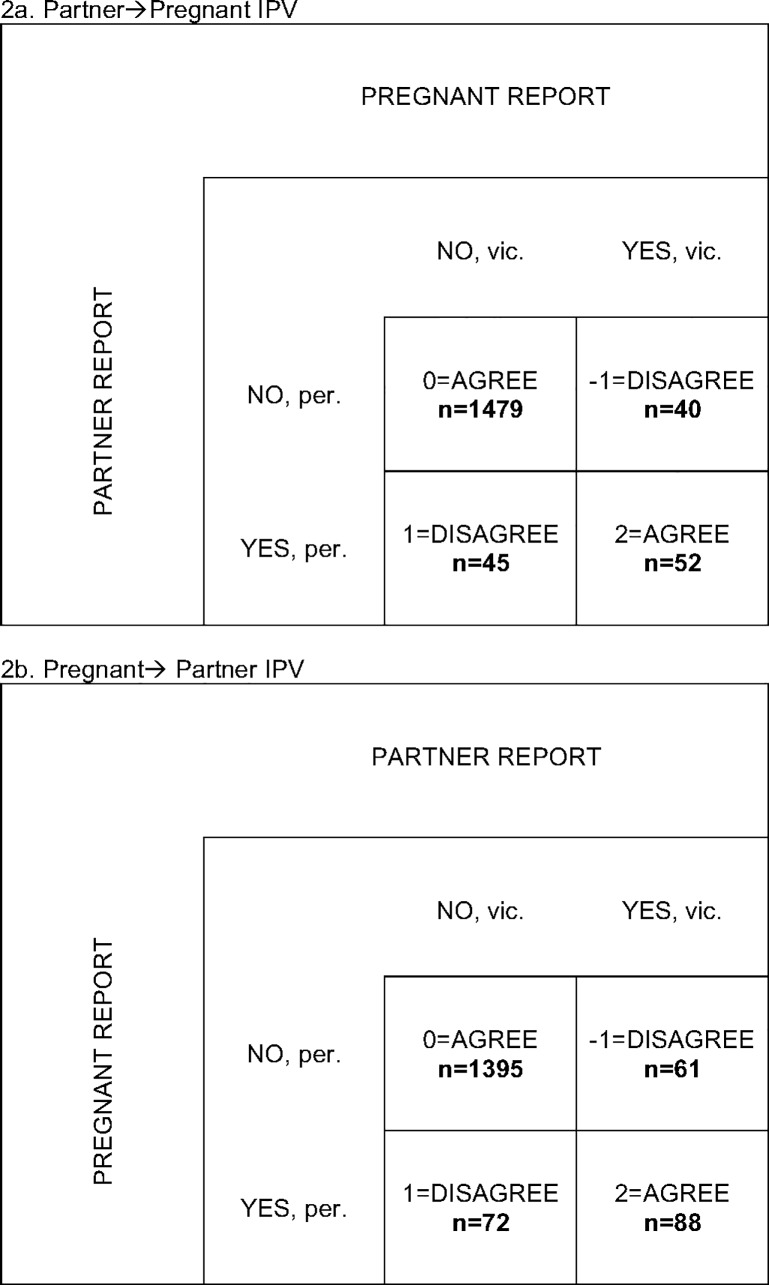
The concordance of pregnant and partner report on physical IPV.

The concordance of reports (Fig 2A and 2B) were equally high for pregnant-woman-to-partner and partner-to-pregnant-woman physical IPV, indicating no gender bias in reporting on these acts. This finding was similar to previous findings [38]. As shown in Fig 2A, intradyadic agreement was 88.7% and 85.9% for partner-to-pregnant-woman and pregnant-woman-to-partner reports, respectively. Notably, in cases with disagreement, the number of reports from pregnant perpetrators (n = 72) was higher than the number of reports from partner victims (n = 61).

## Discussion

Results showed that a substantial number (18.5%) of the expectant couples in this sample experienced some type of IPV during the past year. About 8% of all couples experienced either psychological or physical IPV of a severity that met or exceeded the ICD-11 threshold for clinically significant harm. Furthermore, our findings suggest that, among couples with physical CS-IPV, this violence was as often perpetrated by the pregnant woman as by the partner. In cases of physical LI-IPV and psychological CS-IPV, the violence was more often perpetrated by the pregnant woman. Given that almost no studies—in the Nordic region or elsewhere—investigating IPV perpetrated by pregnant women, this is an important and sobering finding for prevention planners.

### The prevalence of IPV

The prevalence of IPV seen in the current sample was surprisingly higher than the rates of 1.8–5.0% reported in previous studies of Nordic samples on past-year violence against pregnant women [31,29,28]. This may be explained by the fact that we used a more comprehensive survey method and reports from both partners. In general, detailed and comprehensive questionnaires are required to measure the magnitude and dimensions of IPV. For example, more respondents will confirm a statement such as “my partner pushed or shoved me 1–3 times during the past 12 months” as opposed to “my partner has physically abused me”. However, when the prevalence rates of the current study are compared with those seen in pregnant samples from lower-to-middle income countries in South Asia, East Asia and Africa (2–35% for physical IPV and 22–65% for psychological IPV), they are still relatively low [3]. It is important to mention that the large variations in prevalence rates between and within low- and high-income regions likely reflect both the cultural specificity of family violence and differences in study methodology.

### The type and severity of IPV

Typological approaches have been highly influential in understanding IPV. Johnson [39] used the term *situational couple violence* to describe violence that occurs when contextual stressors result in couple conflicts to escalate into IPV [39–41]. According to Johnson’s typology, situational couple violence is more often bi-directional or perpetrated by women, rarely severe, and less likely to persist or escalate over time. This makes it distinguishable from *intimate terrorism*, which is primarily—though not solely—perpetrated by men and motivated by a desire to maintain dominance and control over their partners. Intimate terrorism is, according to the typology, more likely than situational violence to escalate (in frequency and severity) over time and to result in severe injury and fear in the victim. In the current sample, less than 1% (15 couples) reported fear for safety caused by physical IPV and less than 1% (12 couples) reported fear for safety caused by psychological IPV. On the one hand, this would imply that situational violence characterizes the majority of IPV indicated in our sample. On the other hand, our sample deviates from Johnson’s description of situational violence in important ways. First, using the threshold from the ICD-11 classifications, results showed that even if the violence experienced by this sample is, for the most part, not life- or safety-threatening, a substantial proportion of IPV results in significant harm and warrants clinical attention. Second, most perpetrators of uni-directional IPV in our sample were women. Third, among couples with physical CS-IPV, one in three experienced bidirectional IPV and this group reported the largest variety of acts, had the highest total number of acts, and reported impacts that were as severe as the groups with uni-directional physical CS-IPV. It may be more useful for prevention planners to approach IPV in other ways than typologically, especially because there is little evidence to support the idea that IPV typologies (i.e., situational violence versus intimate terrorism) are stable over time [42]. Alternatively, Lorber, Xu, Heyman, Slep, and Beauchaine [43] found that patterns of family violence may be better characterized by a gradient of increasing severity that goes along with other psychological health problems, including depressive symptoms and alcohol abuse. To inform prevention, more research is needed to understand how couples with lower- versus higher-impact IPV change over time, particularly during stressful time periods such as the transition to parenthood.

### Strengths and limitations

We obtained a high response rate (97%) at baseline among the couples signing up for parent preparation. Based on demographic characteristics, the current sample is similar to other primary prevention samples reached during pregnancy in other studies [44,45], and so valid inferences can be drawn on IPV in this population. The survey on IPV was completed anonymously and included highly detailed questions on both acts and impacts. This should reduce the risk of underreporting and, in fact, no systematic underreporting of physical IPV perpetration seemed to appear when reports from victims and perpetrators were compared. However, our findings should be considered in the light of methodological limitations. First, our results would need to be replicated both in other Danish samples and in other European and non-European countries. Second, despite the success at recruitment, women with psychological health problems (including substance abuse) were underrepresented in the current sample because referrals to specialized family treatment was made prior to the recruitment for the intervention offered through this study. Previous studies consistently find psychological health problems and substance abuse to be risk factors for and/or sequelae of IPV [2,46]; thus, the prevalence and severity of IPV indicated by this study is most likely lower than would be the case if all pregnant women could be included. Also, among those we did approach, 41% declined the invitation to participate in the larger intervention study. As is the case with all research on IPV, those with the most severe and terroristic IPV are the most likely to not participate in health services and in voluntary studies. Finally, some types of IPV were very infrequent, causing low numbers in some of the cells of Tables 2 and 3. These should be interpreted with caution.

### Implications for intervention

The transition to parenthood may offer a unique window of opportunity during which expectant parents are especially motivated to work on improving the circumstances for their unborn children [47]. For children, exposure to parental IPV is a risk factor for poor emotional, behavioral, social and cognitive functioning [48]. In addition, US data suggest that 95% of parents who engage in IPV also engage in some form of parent-to-child aggression [49]. For this reason, early family-oriented prevention programs targeting IPV may have a great potential in breaking current or future cycles of family violence.

Although crisis protocols and targeted treatments for IPV exist and are necessary, improving public health approaches to IPV should include addressing it before harmful patterns become entrenched. Screening for IPV victimization among pregnant women has been recommended as a universal approach for several years in the USA [50] but not in the Nordic countries.

In our sample, about 1% of couples (15 of 1407) reported fear that their partners presented a threat to their safety, a clear contraindication for dyadic interventions with both partners present ([51]). Research on relationship therapy indicates that those with and without low-level IPV benefit equally ([52]). Thus, 99% of our couples are likely appropriate candidates for prenatal primary prevention, although it will be important to establish both the safety and efficacy of such programs. Messages that promote respectful, non-violent behaviors in intimate relationships can be integrated into routine parent preparation programs. Further, future research could investigate the benefit of helping couples to an increased understanding of their own conflict pattern, including how their typical conflicts are triggered by between-partner differences, external stressors, intense emotional states, and coercive patterns of communication [53].

Health care professionals who are directly involved with families during pregnancy and postpartum period are in a key position not only to detect and report severe cases of IPV, but also to help implement and deliver the tools to prevent such violence from continuing. For this reason, it is essential that health care professionals (a) are aware that IPV does occur among seemingly low-risk couples and (b) are equipped with knowledge, tools and skills to identify and understand IPV and (c) know of available health services, prevention services, and treatment options so that they can assist their patients to further care.

## Conclusion

Preventing IPV is both a challenge and a high priority within public health approaches across the globe. Multiple approaches must be involved to address the issue appropriately. The current results highlight the fact that couples who are otherwise considered low-risk during their first pregnancy may still be affected by IPV and that, even in this population, the issue causes clinically significant harm to both the pregnant woman and her partner. This knowledge can be of use to planners of routine approaches to pre- and postnatal care. More research needs to be undertaken to understand the developmental course of lower- and higher-impact IPV and to test potential tools or interventions targeting IPV that can be integrated in routine pre- and post-natal care.

## Supporting information

S1 Table (DOCX)
