# Supplementary material for: The prevalence of intimate partner violence among couples signing up for universally offered parent preparation
Source: PLoS One. 2019 Oct 15;14(10):e0223824. doi: 10.1371/journal.pone.0223824 (PMC6793941; doi:10.1371/journal.pone.0223824)
Supplement: S1 Table — (DOCX) [file pone.0223824.s001.docx]

| **S1 Table. Comparison of study participants and the general population of first time parents in Aarhus** | | | | | | | | |
| --- | --- | --- | --- | --- | --- | --- | --- | --- |
|  | Study participants | | | First time parents in Aarhus. 2014 | | |  |  |
| Variables | # Observations | Mean | Std. Dev. | # Observations | Mean | Std. Dev. |  | *T* value |
| Women |  |  |  |  |  |  |  |  |
| *Personal background characteristics* |  |  |  |  |  |  |  |  |
| Age at first birth | 1317 | **29.47** | 3.69 | 3088 | 30.4 | 4.75 |  | -7.00115 |
| Immigrant (0/1) | 1317 | **0.065** | 0.246 | 3088 | 0.170 | 0.376 |  | -10.963 |
| Wage income (in 1000 DKK) | 1317 | **176.2** | 148.6 | 3088 | 216.7 | 169.6 |  | -7.93026 |
| Share of year unemployed | 1317 | 0.038 | 0.112 | 3088 | 0.042 | 0.127 |  | -1.04159 |
|  |  |  |  |  |  |  |  |  |
| Educational background (completed): |  |  |  |  |  |  |  |  |
| High school or less | 1297 | 0.371 | 0.483 | 2903 | 0.351 | 0.477 |  | 1.244554 |
| Short or medium length further education | 1297 | 0.443 | 0.497 | 2903 | 0.415 | 0.493 |  | 1.691018 |
| Long further education | 1297 | **0.186** | 0.389 | 2903 | 0.235 | 0.424 |  | -3.66655 |
|  |  |  |  |  |  |  |  |  |
| *Mood and mental health* |  |  |  |  |  |  |  |  |
| Any psychiatric contacts before 2014 (0/1) | 1317 | **0.072** | 0.259 | 3088 | 0.104 | 0.306 |  | -3.54992 |
|  |  |  |  |  |  |  |  |  |
| Men |  |  |  |  |  |  |  |  |
| *Personal background characteristics* |  |  |  |  |  |  |  |  |
| Age at first birth | 1271 | **31.16** | 4.71 | 2950 | 32.41 | 5.59 |  | -7.46397 |
| Immigrant (0/1) | 1271 | **0.057** | 0.233 | 2950 | 0.172 | 0.378 |  | -12.0454 |
| Wage income (in 1000 DKK) | 1271 | **234201** | 199036 | 2950 | 288550 | 227007 |  | -7.79305 |
| Share of year unemployed | 1271 | 0.028 | 0.103 | 2950 | 0.034 | 0.119 |  | -1.65475 |
|  |  |  |  |  |  |  |  |  |
| Educational background (completed): |  |  |  |  |  |  |  |  |
| High school or less | 1245 | 0.426 | 0.495 | 2771 | 0.459 | 0.498 |  | -1.95028 |
| Short or medium length further education | 1245 | **0.369** | 0.483 | 2771 | 0.310 | 0.462 |  | 3.628393 |
| Long further education | 1245 | 0.206 | 0.404 | 2771 | 0.231 | 0.422 |  | -1.78862 |
|  |  |  |  |  |  |  |  |  |
| *Mood and mental health* |  |  |  |  |  |  |  |  |
| Any psychiatric contacts before 2014 (0/1) | 1271 | **0.043** | 0.204 | 2950 | 0.072 | 0.258 |  | -3.8995 |

Note: Bold indicates that mean among study participants is significantly different from first time parents in Aarhus in 2014 at a 5 % significance level. Wage income is measured in 2013, while unemployment status and completed education are measured in 2014.
